# Review on the Computational Genome Annotation of Sequences Obtained by Next-Generation Sequencing

**DOI:** 10.3390/biology9090295

**Published:** 2020-09-18

**Authors:** Girum Fitihamlak Ejigu, Jaehee Jung

**Affiliations:** Department of Information and Communication Engineering, Myongji University, Yongin-si 17058, Gyeonggi-do, Korea; girumfitex@gmail.com

**Keywords:** structural annotation, functional annotation, ab initio annotation, homology-based annotation

## Abstract

**Simple Summary:**

Due to the development of high-throughput sequencing technologies, computational genome annotation of sequences has become one of the principal research area in computational biology. First, we reviewed comparative annotation tools and pipelines for both annotations of structures and functions, which enable us to comprehend gene functions and their genome evolution. Second, we compared genome annotation tools that utilize homology-based and ab initio methods depending on the similarity of sequences or the lack of evidences. Third, we explored visualization tools that aid the annotation process and stressed the need for the quality control of annotations and re-annotations, because misannotations may happen due to experimental errors or missed genes by preceding technologies. Finally, we highlighted how emerging technologies can be used in future annotations.

**Abstract:**

Next-Generation Sequencing (NGS) has made it easier to obtain genome-wide sequence data and it has shifted the research focus into genome annotation. The challenging tasks involved in annotation rely on the currently available tools and techniques to decode the information contained in nucleotide sequences. This information will improve our understanding of general aspects of life and evolution and improve our ability to diagnose genetic disorders. Here, we present a summary of both structural and functional annotations, as well as the associated comparative annotation tools and pipelines. We highlight visualization tools that immensely aid the annotation process and the contributions of the scientific community to the annotation. Further, we discuss quality-control practices and the need for re-annotation, and highlight the future of annotation.

## 1. Introduction

Next-Generation Sequencing (NGS) has facilitated the generation of vast amount of DNA sequence information from a broad array of lifeforms in amazingly short time [1]. However, information stored in each sequence needs to be extracted, to help us understand the organism itself and evolution in general. NGS has also made possible the investigation of the genetic bases of diseases and gene mapping through large scale screening of genome variation. Therefore, this information benefits processes such as genetic disorder diagnosis and drug design [2]. Annotation is a means of retrieving information encoded within the multitude of different sequence patterns of the four nucleotides (i.e., A, T, C and G). The term genome annotation has evolved from the annotation of protein-coding genes to include the annotation of single nucleotides on thousands of individual genomes. A successful annotation depends on the quality of the genome assembly. Several statistical methods are employed to describe the completeness and contiguity of an assembly [3]. Improvements in sequencing, including long-read [4] and linked-read [5] technology have made high-quality genome assemblies available at lower prices. The availability of high-quality genome assemblies has provided a robust source for phylogenetic information, and this, in turn, has been leveraged to improve whole-genome alignments and annotations, which have heavily relied on models, from mice and humans [6].

Finding and identifying genes constitutes a big part of genome annotation. Therefore, a comprehensive and accurate gene discovery approach is crucial. It entails the application of multiple independent and complementary analysis tools and methods. The employed approaches should hence utilize information that is intrinsic, e.g., ab initio predictions, and extrinsic, including information on proteins and transcripts. Numerous software tools and methods have been developed to tackle the various problems associated with annotation, but the challenge continues, as the technology develops and the knowledge grows [7,8,9].

**Figure 1:**
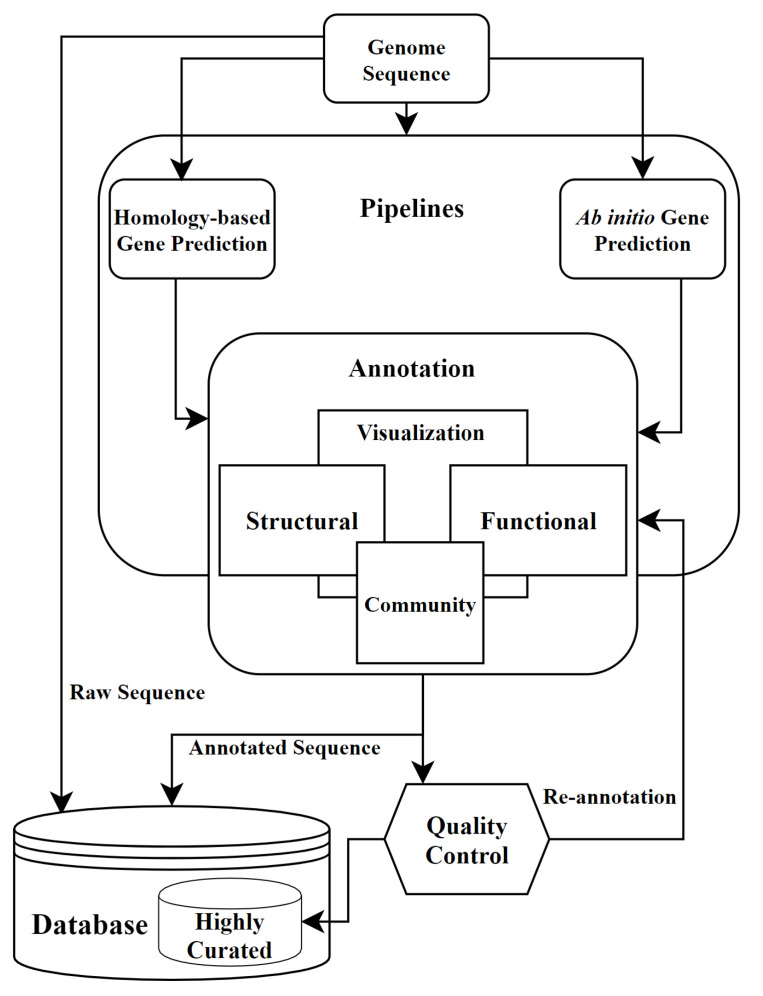
Genome annotation workflow.

In this paper, we summarize the current definitions and tools used for genome annotation. Most genome annotation tools require different types of input formats, and provide various types of outputs. Depending on the research environment, researcher can choose one of applications. We start by highlighting the structural and functional annotation processes, and commonly used programs. The goals of structural and functional annotation can be achieved through the analysis of sequence data, by exploiting either a statistical model approach (the ab initio method) or a sequence similarity technique (homology-based annotation), although these approaches are not mutually exclusive. In parallel, databases that play an integral part in annotation will be discussed. Annotation pipelines that aggregate ab initio and homology-based methods, together with other software components to generate well-annotated genomes, are presented. Even though our focus in this review concerns sequence annotation, we discuss how annotation plays a major role in identifying a potential disease-causing gene or causal mutation. Therefore, databases and tools used for gene variant annotation are described as well. Annotation is not an easy task and visualization tools are useful in facilitating it. We hence explore different genome browsers that are used for gene structure and function predictions, as well as other visualization tools that aid the analysis of gene function. The annotation data should undergo quality checks, as errors can be easily propagated, and affect downstream annotation and analysis. A quality-check result may necessitate genomic re-annotation, which may go as far as re-sequencing, sometimes discarding the original version. We conclude by examining the future possibilities of annotation. The entire annotation workflow following sequence assembly is summarized in Figure 1. This workflow can also be considered as a graphic summary of this review.

## 2. Types of Annotation

### 2.1. Structural Annotation

Finding features of DNA—exons, introns, promoters, transposons, etc.—is known as structural annotation. While structural annotation attempts to find genes in a genomic sequence, gene definition has evolved with the advances in modern genomics. A gene can be defined as "a sequence region necessary for generating functional products" [10]. Functional products of genes are proteins and RNAs. Genes that lead to the production of proteins are called protein-coding genes. Other genes that do not code proteins, but instead functional RNA molecules, are called noncoding genes. Noncoding RNA genes include genes for ribosomal RNA (rRNA), transfer RNA (tRNA), microRNA (miRNA), small nuclear RNA and nucleolar RNA (snRNA and snoRNA, respectively) [11] and long noncoding RNA (lncRNA). Structural annotations also identify pseudogenes. They were initially considered to be functionless and evolutionary dead-ends. We now know that they sometimes participate in gene regulation [12]. Hence, their prediction improves our understanding of genomes.

#### 2.1.1. Repeats

The first step in structural annotation involves repeat masking. DNA repeats occur in both prokaryotic and eukaryotic organisms. The repeats account for 0% to over 42% of the prokaryotic genome [13]. Similarly, eukaryotic genomes can harbor millions of repeats. For instance, repeats account for two-thirds of the human genome [14]. Repeat sequences can be localized in tandem, i.e., adjacent to one other, and are typically found in the centromere [15]. Alternatively, they can be interspersed in different forms of transposable elements, e.g., in long and short interspersed nuclear elements (LINEs and SINEs), DNA transposons, etc. [16]. Identification of the essential features of repetitive elements is still challenging, despite advances in repeat identification. Repeat masking tools rely on databases with lists of already identified repeats. RepeatMasker [17] is a good example of such tool.

Aligning transcript and protein evidence after masking is the second step of structural annotation before gene identification, although it is not mandatory. BLAST [18] or BLAT [19] can be used to align the transcript and protein evidence. Further, RNA-seq evidence can be aligned using TopHat [20] or HISAT [21].

#### 2.1.2. Predictions of Gene and Different Features

Identifying protein-coding genes and other regulatory elements takes center stage in gene annotation. Gene prediction is a complex process, especially for eukaryotic DNA [3]. The varying sizes of introns (noncoding sequences) in-between exons and alternative splice variants make gene structure prediction difficult. Many gene prediction programs exist. They can be categorized into three groups: ab initio methods, homology-based methods, and combined methods. Approaches for gene prediction based on nucleotide sequence are called ab initio methods. Ab initio approaches rely on statistical models, such as the hidden Markov model (HMM), to identify promoters, coding or noncoding regions, and intron–exon junctions in the genome sequence. The second approach aligns the sequence with expressed sequence tags (EST), complementary DNA (cDNA), or protein evidence, and uses detected similarities for gene prediction. The other group comprises programs that combine ab initio and evidence- or homology-based approaches for gene prediction [22]. In addition, gene prediction programs should be able to predict alternative splicing sites because alternative splicing is a major actor in the regulation of gene expression, and transcriptome and proteome diversity [23]. Accordingly, gene prediction programs use various models to predict splice sites. Since approximately 99% of the introns in sequenced genomes begin with GT and end with AG, these features are denoted as mandatory by most gene prediction systems for splice site detection. In addition, incorporation of a strong splice donor consensus, such as the GC–AG splice site, improves the accuracy of gene prediction programs [24]. Commonly used gene prediction programs and their classification, based on the above discussion, are listed in Table 1.

#### 2.1.3. Databases for Structural Annotation

Annotations require supporting data that can be used or presented as evidence of predicted assignments. Currently, homology-based methods play a central role in genome annotation because of the huge amount of EST and cDNA sequences available [44]. Homology-based methods depend on DNA, RNA, or protein sequence alignment data, which can easily be retrieved from biological databases. Ab initio annotations, on the other hand, identify genes and their structures using mathematical models. Nonetheless, the ab initio gene predictors have to be trained using high-quality gene models or organism-specific genome traits, such as codon frequency and intron–exon length distribution [45]. Further, ab initio models require ESTs, RNA-seq data, and proteins to improve prediction accuracy. Databases readily provide such data.

Nucleotide and protein sequence or structure can easily be found in comprehensive public-domain databases, e.g., the GenBank [46], European Nucleotide Archive (ENA) [47], and DNA Databank of Japan (DDBJ) [48]. UniProt [49], which is a protein sequence database that combines UniProtKB/Swiss-Prot (over 560,000 manually curated sequences) and UniProtKB/TrEMBL (180 million automatically annotated sequences), provides the scientific community with high-quality and freely accessible protein sequences with the associated functional information. Another great database for protein annotation is InterPro [50], which provides information on protein families, domains, and important sites such as binding sites, active sites, conserved sites, and repeats. The InterPro Consortium has 14 member databases, including Pfam [51], PROSITE [52], TIGRFAM [53], CATH-Gene3D [54], and PANTHER [55].

In addition, some specialized databases are built as comprehensive one-stop points of information on specific topics of interest. For example, databases, such as NONCODE [56], Pseudogene.org [57], Dfam [58], and miRbase [59] have contributed to the structural annotation of noncoding RNAs, pseudogenes, transposable elements, and microRNAs, respectively.

### 2.2. Functional Annotation

Association of biological information with gene or protein sequences identified by structural annotation is called functional annotation. Protein-coding genes were the focus of traditional functional annotation. However, the many different functions of noncoding genes and untranslated transcripts are currently recognized. The term "functional" has a very different meaning to evolutionary biologists, who are interested in conservation, and experimental biologists, who are interested in biochemical roles [60]. However, the basic portion of functional annotation involves the association of a functional description with a gene, after identifying a similar sequence using tools, such as BLAST.

Functional annotation is also employed to assess the variation in genes. Annotation of genomic variants is an increasingly important and complex part of the analysis of sequence-based genomic analysis. The goal of variant annotation is to identify and prioritize variants based on their functional impacts [61]. Molecular impacts of genetic variants on phenotypes can be understood by assigning structural and functional knowledge of genomic sequences to variants [62]. Gene variation can happen because of a single nucleotide change in between members of same biological species’ genomic DNA, called a single nucleotide polymorphism (SNP), or by structural rearrangements such as insertion, deletion, translocation, and inversion. Insertion and deletion cause variation known as copy number variations (CNVs) [63]. The functional relevance of variants can be explored in databases that have functional annotation information of known and novel variants.

#### 2.2.1. Automatic Functional Annotation

Although manual annotation is still considered as the gold standard, this approach is difficult to scale. This necessitates the use of automated annotation methods, to scale up to match the plethora of genomic data currently being generated with NGS technology. Automatic function prediction can be achieved directly, by using local alignment tools, such as BLAST, where a protein database is searched for high-scoring alignments. The function is then assigned to the unknown query sequence based on a known result sequence, provided that it is the highest scoring alignment from all sequences, above some specified threshold value. The assumption of function transfer on which BLAST-like tools rely is that the function is retained in proteins that have similar sequences and have evolved from a single ancestor. In other words, the tools identify evolutionary relationships by discovering orthologous and paralogous relations between sequences. Orthologs are genes that have originated from a single ancestral gene in the last common ancestor of the compared genomes, whereas paralogs are genes within the same genome that have arisen from duplications [64]. Local alignment-based functional annotations are simple to use and perform well in many cases. However, they have some drawbacks. Examples include database source error, relativity of the alignment threshold, low sensitivity/specificity, and excessive transfer of annotation from an unrelated local region of similarity [65].

#### 2.2.2. Databases for Functional Annotation

#### Gene Ontology (GO)

The GO resource is the most comprehensive and widely used knowledgebase for gene function [66]. GO covers three aspects of gene function: the molecular function (activity of a gene product at the molecular level), the cellular component (location of the gene product), and the biological process (a biological program, in which a gene’s function is used). Gene products (proteins and RNA) should be consistently described to allow a comprehensive coverage of biological concepts. The GO Consortium tries to address this need by developing and maintaining ontology standards, annotating gene products, and developing and maintaining tools to do so [67]. The standard GO annotation comprises gene, GO term, and scientific evidence elements. These only reflect a partial functional description because single GO term annotations represent minimal knowledge determined from few experiments. Hence, a model concept called GO Causal Activity Modeling (GO-CAM) was introduced in 2018 to extend the existing annotation to represent a complex statement that can be scalable and structured [68]. Multiple standard GO annotations are linked to larger models of biological functions by GO-CAM in a semantically structured manner. The explicit relationship between the molecular function, biological process, and cellular component of each gene function is also defined by GO-CAM. This results in improved quality and consistency of GO annotations [69]. Additional functional databases, other than the GO database, exist. Nonetheless, the GO database is quite popular and different tools have been developed to use its rich ontologies for annotation. Table 2 lists useful functional annotation tools.

Kyoto Encyclopedia of Gene and Genomes (KEGG) [76] acts as a link between genomic data and higher-order functional information, which are stored in the GENES and PATHWAY databases, respectively. It affords understanding of high-level functions and utilities of the biological system, such as the cell, organism, and ecosystem, from genomic- and molecular-level information. The KEGG Orthology (KO) database links genes to high-level functions [77]. The KO system is the basis for genome annotation and KEGG mapping, which replaced the EC number that linked genomes to metabolic pathways.

The Reactome Pathway Knowledgebase [78] focuses on *Homo sapiens*, linking human proteins to their molecular processes. The Reactome data model presents molecular details and processes of signal transduction, transport, DNA replication, and metabolism as an ordered network of molecular transformations. Reaction is the core unit in the Reactome data model. Nucleic acids, proteins, and other molecules participate in reactions, forming a network of interactions, and are grouped into pathways, such as metabolism, regulation, and disease. The recent addition of a new drug class to the database extended the annotation process to human diseases [79].

Rhea [80] enables the functional annotation of enzymes and description of metabolic pathways based on an expert-curated non-redundant resource of biochemical reactions.

ChEBI [81] contains manually curated data of chemical entities that are classified into two sub-ontologies. The chemical entity ontology classification is based on common structural features and the role ontology considers activities in biological and chemical systems, or applicability.

NCBI’s Conserved Domain Database (CDD) [82] comprises protein domains conserved during molecular evolution, and provides conserved domain footprints, along with conserved functional site annotations of protein sequences. Evolutionarily conserved domains help transfer the functional annotation from a known domain model to protein sequence. The Conserved Domain Architecture Retrieval Tool (CDART) groups proteins into superfamilies, while the Subfamily Protein Architecture Labeling Engine (SPARCLE) groups them according to subfamily domain architectures.

The Database of Genomic Variants (DGV) [83] provides a comprehensive summary of genomic variations (structural variations) that are larger than 50 bp (base pairs) from DNA segments of the human genome. It contains structural variations identified in healthy control samples and provides a useful catalog of control data for studies aiming to correlate genomic variation with phenotypic data.

dbVar [84] is a human genomic structural variation database from the NCBI. It contains more than six million submitted structural variants and has the same data model as DGV that allows the implementation of standardized terminology. For the functional analysis of SNPs, the NCBI also provides another database called the dbSNP [85].

The Human Gene Mutation Database (HGMD) [86] constitutes information about gene mutations associated with human inherited disease and functional SNPs.

HGVbase [87] is a human sequence variation database that has high quality and non-redundant variation data and it mostly comprises SNPs.

The International Genome Sample Resource (IGSR) [88] is an extension of the 1000 Genomes Project [89] data, which served as a reference set of human variation. It maintains and updates to 1000 Genomes Project resources to the GRCh38 (Genome Reference Consortium human assembly). The web-based portal includes samples that were not part of the 1000 Genome Project and presents a unified view of data from multiple studies.

## 3. Comparative Annotation Methods

Genome annotation achieved by comparison of genes and genomes across species can be a reliable information source for understanding genome evolution. Comparative annotation allows annotations of a well-studied genome to be projected onto an evolutionarily close species. It often focuses on the coding genes. Valuable information for comparative annotation can be found from genome alignment. A well-aligned genome will yield sound data for comparative annotation [90]. Approaches to comparative annotation of genomes can be categorized into ab initio methods and homology-based methods, considering the input information used for annotation, i.e., either a statistical model of genes, or protein sequence, EST, and cDNA, accordingly. Ab initio approaches are preferred for genes that are weakly or not at all represented in RNA-seq library and have insufficient similarity to any known protein and lack other evidence.

Several comparative annotation methods have also been developed for variant calling purposes [91]. Like sequence annotation, variant calling annotation starts with alignment against a reference genome. Various tools exist to perform the variant calling and they produce a variant calling format (VCF) file for further downstream analysis.

### 3.1. Ab Initio Annotation

Ab initio annotation relies on ab initio gene predictors, which in turn rely on training data to construct an algorithm or model. Prediction is done based on the genomic sequence in question, using statistical analysis and other gene signals such as k-mer statistics and frame length. Some popular ab initio gene predictors are discussed below.

AUGUSTUS [42] defines the probability distributions for eukaryotic genome sequences based on GHMM. AUGUSTUS is re-trainable and it can predict alterative splicing, and the 5′UTR and 3′UTR, including introns. AUGUSTUS is one of the most accurate ab initio gene prediction programs for the species it has been trained for [92].

FGENESH [93] is an HMM-based, very fast, and accurate ab initio gene structure prediction program for humans, *Drosophila*, plants, yeasts, and nematodes. When applied to single-gene sequences, FGENESH predicts approximately 93% of all coding exon bases, as well as 80% of human exons, in 1.5 min. This renders it the fastest tool among HMM-based gene finding programs [26].

GENSCAN [29] is another HMM-based ab initio tool for predicting locations and exon–intron structures of genes in genomic sequences of a variety of organisms. Vertebrate and invertebrate versions of GENSCAN are available. The accuracy of the latter is lower because the original tool was primarily designed for the detection of genes in human and vertebrate genomic sequences.

It is becoming a common practice to use ab initio annotation methods in combination with transcriptome information such as that provided by RNA-seq. [60], particularly for higher eukaryotes. This can be viewed as an evidence-based or extrinsic approach. For example, a newer version of AUGUSTUS can incorporate information from EST and protein alignments. In addition, a variant of FGENESH called FGENESH-C [94] uses HMM and cDNA for predictions, while GenomeScan (an extension of GENSCAN) uses extrinsic information of protein BLAST alignments [95] for gene structure prediction.

### 3.2. Homology-Based Annotation

According to the molecular evolution principle, the rate of evolution of functionally important portions of the genome is slower than the rest of the cellular molecular regions. Hence, gene sequences that are useful for survival and other crucial functions are conserved [96], especially in closely related species. Homology-based annotations exploit this fact, to predict and annotate genes by identifying significant matches from a well annotated genome sequence by employing alignment tools such as BLAST. Homology-based annotations use the coding sequences (CDS), usually protein sequences and sometimes transcripts in the form of mRNA, cDNA, or EST to predict genes, assuming similar sequence regions encode homologous proteins. Tools like Exonerate [97] and DIALIGN [98] can be used for sequence alignment; GenomeThreader [38] and AGenDA [99] are used for gene predictions. Increased evolutionary distance between the input protein and the target protein reduces the accuracy of homology-based gene finding [41]. This happens because of heavy reliance on the alignment and information derived from the already known genes, which creates a challenge in identifying genes whose properties are different from those of referenced genes. However, newer comparative approaches solve this issue by relying to a greater degree on sequence conservation, which enables them to identify genes with new features and different statistical composition. TWINSCAN [41] and SGP2 [100] are examples of tools in which gene prediction uses the analysis of sequence conservation patterns between genomic sequences of evolutionarily related organisms [101]. Additional gene predictors used for homology-based annotations are listed in Table 1.

Table 3 below summarizes and compares ab initio and homology-based annotations discussed above.

### 3.3. Variant Annotation

SnpEff [102] annotates and predicts the effects of variants on genes. It categorizes the effects of SNPs and other variants such as multiple nucleotide polymorphisms (MNPs). SnpEff accepts predicted variants in VCF and annotates the variants plus the effects (e.g. amino acid changes) they produce on known genes.

Ensembl Variant Effect Predictor (VEP) [103] performs annotation and analysis on genomic variants of coding and noncoding regions. It is a flexible tool for the identification of genes and transcripts affected by variants along with their location.

GEMINI [104] is a framework that allows exploring all forms of human genetic variation. GEMINI integrates genetic variation with diverse genome annotation from databases such as dbSNP and KEGG. It accepts a VCF file automatically and annotates by comparing with annotation sources.

SeattleSeq [105] provides annotation of SNVs and small indels, both known and novel. The annotations include dbSNP reference tags, gene names and accession numbers, variation functions, protein positions and amino acid changes, conservation scores, and clinical associations.

SNPnexus [106] is a web-based solution for functional annotation of novel and public domain variations. It allows assessing the potential significance of variants from broad range of annotation categories.

## 4. Annotation Pipelines

Analysis of large amounts of data generated by the NGS requires multiple computationally-intensive steps [107]. Sets of algorithms that process NGS data and are executed in a predefined order are called a bioinformatic pipelines. Pipelines process massive amounts of sequence data and the associated metadata using multiple software components, databases, and environments [108]. They are comprehensive, holistic packages that try to exploit relevant information provided by both ab initio and similarity-based gene predictors.

### 4.1. Structural Pipelines

MAKER2 [109] is a multi-threaded, parallelized genome annotation and data management application, which builds up on MAKER [110]. MAKER2 is designed for second-generation genome projects, which lack pre-existing gene models to train gene finders, but it also performs well with first-generation genome projects. Ab initio gene prediction tools SNAP, AUGUSTUS, and GenMark-ES are integrated in MAKER2. Novel genomes with limited training data available can be annotated with MAKER2. The tool can also be used to improve annotation quality by integrating mRNA-seq data. Further, it can be used to update legacy annotations.

NCBI Eukaryotic Annotation Pipeline [111] is an automated pipeline for eukaryotes, in which coding and noncoding genes, transcripts, and proteins in both finished and draft genomes can be annotated. This pipeline uses Splign [112] and ProSplign for alignment. It also has its own gene prediction tool called GNOMON which combines HMM-based ab initio models and homology search information extracted from experimental evidence.

Comparative Annotation Toolkit (CAT) [113] is a fully open-source software toolkit for end-to-end annotation. CAT uses Progressive Cactus [114] for multiple alignments. Its output, together with previously annotated genomes, is used to project annotations using TransMAP [115]. CAT uses AUGUSTUS for gene prediction both from transMap projections and for ab initio gene prediction, integrating extrinsic information from RNA-seq and Iso-Seq transcripts. All sources of transcript evidence are combined in an annotation set using a consensus-finding algorithm within CAT. CAT was developed by the GENCODE [116], and was utilized for the annotation of genomes of laboratory mouse strains [117] and great apes [118].

BRAKER1 [119] is a fully automated and highly accurate unsupervised RNA-seq–based genome annotation pipeline for eukaryotic genomes. It merges the complementary strengths of GeneMark-ET [120], which generates initial ab initio gene structure predictions via unsupervised iterative training of unassembled RNA-seq reads, and AUGUSTUS, which uses the predicted genes as a training set, to predict genes, utilizing mapped unassembled RNA-seq read information. BRAKER1-based gene predictions are more accurate than those of MAKER2, when RNA-seq data only are used. BRAKER1 has been expanded to integrate cross-species proteins, along with RNA-seq and protein alignment data, as heterogeneous extrinsic evidence. Furthermore, it is capable of whole-genome annotation [121].

### 4.2. Functional Pipelines

Prokka [122] is Unix-based command line software that can be used for rapid annotation of prokaryotic genomes. It identifies the coordinates of genomic features within contigs using external feature prediction tools, such as RNAmmer and Prodigal [123]. Prokka uses a hierarchical method for annotation, starting with a smaller trustworthy database; then, it moves to a medium-sized domain-specific database, and finally to curated models of protein families, including Pfam and TIGRFAMs.

Rapid Annotations using Subsystems Technology (RAST) [124] is a fully automated pipeline for bacterial and archaeal genome annotation. It achieves accuracy, consistency, and completeness by utilizing a library of subsystems, which are functional roles (abstract protein function) that implement a specific biological process or structural complex [125], together with protein families derived from subsystems. Gene function assertions are made based on both subsystem recognition of functional variants called “subsystem-based assertions” and integration of evidence from different tools called “nonsubsystem-based assertions”.

### 4.3. Combined Pipelines

NCBI Prokaryotic Genome Annotation Pipeline (PGAP) [126] is an aggregation of alignment-based methods with a specialized search tool and ab initio gene finding tool called GeneMarkS+. GeneMarkS+ (a new self-training gene finder that is an extension of GeneMarkS [127] developed for use in PGAP) integrates alignment-based protein predictions, RNA predictions, and other extrinsic information with intrinsic information on genome-specific sequence patterns of protein-coding regions. PGAP uses statistical gene predictions when external evidence is insufficient and capitalizes on sequence similarity if enough comparative data are available.

DFAST [128] is a prokaryotic genome annotation pipeline that supports genome submission to the public database DDBJ. DFAST uses GHOSTX algorithm [129] for homology search with referenced databases. LAST [130] (an adaptive local alignment tool that enables fast and sensitive comparison of large sequences) is used for pseudogene detection by re-aligning CDSs with their subject protein sequences. An additional tool called hmmscan [131] searches for profile HMMs against TIGRFAM database. Clusters of Orthologous Groups (COG) from NCBI are searched for the assignment of COG categories using RPS-BLAST [132]. The DFAST workflow supports both structural and functional annotations, which are implemented as a module with common interfaces allowing flexible annotation.

Genome Sequence Annotation Serve (GenSAS) [133] is an online pipeline that provides both structural and functional annotations for eukaryotic and prokaryotic genomes. In addition to annotations, the GenSAS pipeline enables repeat identification and masking, evidence alignment, optional manual editing of gene models, and creation of final annotation files. It uses more than 25 tools for gene prediction, alignment, and annotation, and integrates some genome browsers.

### 4.4. Variant Pipelines

ANNOVAR [134] annotates SNPs and CNVs and examines their functional consequences on genes. It also performs genomic region-based annotation and compares variants to variation databases. ANNOVAR can evaluate and filter out variants against user dataset or variants that are not reported in public databases. To address personal genome annotation by biologists and clinicians, a web server called wANNOVAR [135] was developed using ANNOVAR as a backend annotation engine.

AnnoGen [136] allows the annotation of chemical binding energy, sequence information entropy, and homology score features for the GRCh38 framework.

Annotatr [137] provides genomic annotations and set of functions to read, intersect, summarize, and visualize genomic regions in the context of genomic annotations. It is a Bioconductor package that provides insights into how characteristics of the region differ across annotations.

## 5. Annotation Visualization

### 5.1. File Formats

Most bioinformatic tools use the FASTA format as a standard for sequence data sharing. The FASTA format is used for searching sequence databases, evaluating similarity scores, and identification of periodic similarity scores. The format can also be used to compare a protein sequence with information in a DNA sequence database, with the DNA database translated while the search is performed [138]. Nevertheless, FASTA is a simple data file format that cannot handle all the information that might be added in the course of an annotation. Other standard file formats exist that can accommodate additional information and can be used by different programs, and interpreted by human users. The most common of these are the GenBank file format of NCBI, DDBJ format of DDBJ, EMBL format of ENA, and general feature format (GFF) and GTF.

The GFF [139] especially has become the de facto reference format for annotations. It stores genomic features in a standard text file format. Its new extended GFF3 format is a nine-column tab-delimited plain text file that addresses deficiencies of the previous versions GFF2/GTF. GFF3 allows flexibility, which enables storage of a wide variety of information. It is widely used for data exchange and genomic data representation.

### 5.2. Genome Browsers

Annotation yields gene structure, gene function, gene expression, regulation, variation, and additional information by employing multiple tools and information sources. Researchers and users utilize genome browsers to integrate various types of information, as well as analyze and visualize data related to annotation. Genome browsers are usually used to efficiently and conveniently browse, search, retrieve, and examine genomic sequence and annotation data, via a graphical interface [140]. They have been deployed since the initial sequence set generated by the Human Genome Project [141]. Although some standalone genome browsers exist, most genome browsers are web-based and can be classified as general and species-specific genome browsers.

General genome browsers host multiple richly annotated genomes from different species and enable comparative analysis. The UCSC Genome Browser [142] is the most commonly used genome browser; many visualization tools are modeled based on this tool. Although its user base focuses on human and mouse research, the UCSC genome browser database, which was founded in 2001, currently hosts more than 105 different species. The Ensembl genome browser [143] is another widely used genome browser for vertebrate genomes, which supports comparative genomics, sequence variation analysis, and transcriptional regulation analysis. The NCBI Genome Data Viewer (GDV), previously known as the Map Viewer, is another browser that supports visualized exploration and analysis of eukaryotic NCBI’s Reference sequence (RefSeq) genome assemblies. Nonetheless, generalized genome browsers cannot handle diverse analyses and the increasing customized visualization in each species-specific area.

Species-specific genome browsers focus on one model organism and help to visualize genomic, epigenomic, and transcriptomics data for that specific organism. As an example, Wormbase [144], Flybase [145], and MaizeGDB [146] provide species-specific browsers, which are based on the GBrowse framework of Generic Model Organism Database (GMOD).

GMOD is a collection of interconnected open-source software tools and databases for managing, visualizing, storing, and sharing genetic and genomic information. The most popular component of GMOD is GBrowse (a generic genome browser) [147], which is a web application for displaying genomic annotations and other features. Customizable design has made GBrowse suitable to act as a building block for databases for many model organisms, including Wormbase, Flybase, and many more. A very fast and scalable successor of GBrowse is the JBrowse genome browser [148]. JBrowse is built with JavaScript and HTML5, and can run standalone analyses or can be embedded in a website. The functionality of JBrowse is greater than that of GBrowse, with greater speed and responsiveness, and click-and-drag navigation, including same-screen track selection. JBrowse is the next-generation of this genome browser, constantly expanded by data migrated from other databases. WebApollo, or simply Apollo [149], is a plugin for JBrowse for viewing and manual annotation of genomes. It allows real-time collaborative editing. Apollo enables concurrent editing by multiple users via WebSockets, which are supported by most web browsers. GMOD software tools have been designed, developed, and tested by many developers, scientists, and laboratories over the years, and have high demands by biologists on account of their interconnectedness. In general, the GMOD project is directed by its user base, who are mostly biologists.

JBrowse was the first genome browser that utilized client-side technology for retrieving and processing data through in-browser JavaScript programming. This enabled the user to cease to entirely rely on a web-server to preprocess data. ABrowse [150] enhanced and extended the interactivity of the JBrowse model by allowing access to more data sources and enabling non-real time commenting and annotation. Likewise, Genome Maps [151], ChromoZoom [152], and PBrowse [153] also try to improve the user experience, with a focus on implementing improved web technologies to handle high-volume data.

### 5.3. Functional Analysis Visualization Tools

Functional annotation of large gene sets (gene lists) is the final step of omics data analysis. It serves for the identification of transcriptional networks, the building of predictive models, and the discovery of candidate biomarkers. This differential analysis is challenging because of the high-dimensional nature of functional gene profiles derived from multiple experiments. Multiple tools are available for graphic representation and analysis of enriched functional annotations. First, explanatory data analysis methods are used to reveal the structure, and then statistical methods are applied to detect biological process patterns. Mapping molecules to biological annotations is a common approach using hierarchical structures of terms from the KEGG pathways, Reactome pathways, and GO terms. The majority of available tools are web services or are implemented in R. We discuss some such tools below.

Database for Annotation, Visualization and Integrated Discovery (DAVID) [154] is a program that facilitates the functional annotation and analysis of large gene lists. DAVID is linked to rich biological annotation sources, which facilitate biological discovery by biochemical pathway mapping, functional classification, and analysis of conserved protein domain architectures. DAVID combines annotation with graphical representation and produces tabular output with query-based access to functional annotation. In addition, DAVID can cluster redundant annotation terms, explore gene names in batch, and execute gene-enrichment analysis, particularly for GO terms.

g:Profiler [155] is a tool for functional enrichment analysis and additional information mining. The web server analyzes gene lists for enriched features, converts different class gene identifiers, maps genes to orthologous genes, and searches similarly expressed genes in public microarray datasets. g:Profiler uses gene annotations and identifiers from Ensembl, and ontologies from the GO website.

GOPlot [156] is an R package for functional analysis that follows deductive reasoning. GOPlot generates a visual representation (plot), from a general identification of most enriched categories to detailed molecule displays, in a specified set of categories.

FunMappOne [157] accepts input of gene lists and modifications and enables a graphical visualization of enriched terms. The output is provided with interactive navigation. Over-represented biological terms from GO, KEGG, or Reactome datasets are evaluated statistically by the functionalities offered by FunMappOne, to graphically summarize and navigate them within super-classes. FunMappOne exploits hierarchical structure of functional annotations of KEGG, GO, and Reactome and homogenizes them to offer three levels of summarization, from terms-root.

Gene Annotation Easy Viewer (GAEV) [158] has been developed to construct the complete set of molecular pathways for non-model species using resources at KEGG, i.e., by integrating KO annotation and KEGG pathway mapping. GAEV software can be run on Windows and Linux machines, and it provides gene function summaries and the association of molecular pathways with genes.

### 5.4. Other Visualization Tools

Visualization plays a major role in displaying the finalized records of organellar (mitochondrial and plastid) genomes. Organellar genomes are the focus of taxonomic relationship studies because they are inherited from single parent and abundant in a cell. These genomes are small, informative, and can be easily sequenced. OGDRAW [159] is currently the standard tool for the generation of graphical maps of organellar genomes. GeneBank file formats are used as an input, and graphical maps of both circular and linear genomes can be drawn using this tool. Visualization of coding regions and other feature-bearing regions, together with gene expression data and cut sites of restriction enzymes can be displayed in OGDRAW. Organellar annotation programs, such as AGORA [160], which uses BLAST-based homology searches for organellar annotation from user or NCBI database reference, and GeSeq [161], an annotator for organellar genomes (particularly for the chloroplast), which identifies genes (by BLAT-based homology search), proteins (by HMM search), and rRNA-coding genes (by de novo prediction), use OGDRAW to visually display the annotation output.

An aesthetically appealing tool called Circos [162] displays genomic interval relationships in a circular ideogram layout. It facilitates the identification and analysis of similarities and differences in large volumes of genomic data. Circos plots are widely used in various genomics studies to demonstrate various genomic data, such as gene rearrangements [163]. However, its use is limited by the installation and command line-based usage. Several tools with different objectives have been developed to address these issues, including CircosVCF [164], MISTIC [165], J-Circos [166], and shinyCircos [167]. Although it is mainly used as a multiple genome alignment tool that identifies conserved regions, rearrangements, and inversions, MAUVE [168] has a simple viewing system that can display structural rearrangements of genomes. The Mauve rearrangement viewer has an interactive feature that enables searching and zooming into regions of interest in aligned genomes. A similar interactive online tool that is used for the display, manipulation, and annotation of phylogenetic trees is the Interactive Tree of Life (iTOL) [169]. This tool can be used to represent phylogenetic trees in several tree formats, including the circular (radial) mode.

## 6. Community Annotation and Quality Control in Annotation

### 6.1. Community Annotation

The advent of NGS technologies has resulted in a large volume of sequencing data and turned the research focus toward genome annotation. Gene databases, such as Ensembl and GenBank, and model organism databases, such as FlyBase provide annotations. However, these sites are authoritative because of the high degree of oversight by expert curators [170]. Continuous sequence annotation is a challenge, especially when only a limited number of professional annotators are available for the databases mentioned above. As an alternative, community engagement helps to deal with annotation bottlenecks. In 2001, Lincoln [171] proposed four models to describe the "sociology of genome annotation." The first model is the so-called "Factory model," which involves a high degree of automated genome analysis for finding genes and identifying structural landmarks. Genome browsers usually use this model. The second model, "museum model," focuses on the functional roles of genes and requires considerable manual input from expert curators, which makes it a good choice for model organism analyses. The third model is the "cottage industry model," and involves decentralized effort from curators at different laboratories. The last model, the "party" or "jamboree model," assembles expert biocurators for a specific time period, usually a week. This model has been famously used for the annotation of *Drosophila melanogaster* [172] and cDNA annotation of the mouse genome [173]. Additional models, the "blessed annotator" and "gatekeeper approach," were presented in 2012 by the Wellcome Trust Sanger Institute [174]. The Blessed annotator is a variation of the Museum approach and is used for the Knockout Mouse Project (KOMP), while the Gatekeeper approach is an extension and refinement of the party and cottage industry models, and is used for the analysis of data for multiple species.

Community annotations can take forms different from the ones described above. For instance, for supervised dispersed-community annotations, experts in a field annotate specific items in response to request from a coordinator [175]. Currently, a community annotation jamboree can take place virtually. One variant of the annotation jamboree is student community annotation, where students are taught during a class or workshop about annotation, and then perform the annotation. This is a mutually beneficial scheme for both the students’ education and genomic resources. Another type of community annotation, which requires the least engagement, is unsupervised dispersed-community annotation or Gene Wiki (Wikis), where anyone can login and annotate an entry of choice, and which is based on the open data model of Wikipedia.

### 6.2. Quality Control for Annotation

Quality of annotation is directly affected by the input genome sequence quality. Although NGS technologies have enabled the generation of sequences in cost effective and short period, they produce reads, ranging from dozens to thousands of consecutive bases, that should be assembled to make a complete sequence. Hence, assessing quality of a sequence assembly is vital before subsequent annotation. Tools such as MaGuS [176], QUAST [177], and BUSCO [178] can be employed to check the quality, contiguity and completeness of genome assemblies.

Manual annotation requires considerable infrastructure and specific tools, which make it costly. Nonetheless, it provides an accurate gene set, which serves as a solid reference for a variety of studies. Manual curation has been held as a gold standard for functional annotation, but newer automatic systems might perform as well as teams of sequence-annotating experts [179].

Automated systems are necessary for meeting the challenge of extracting information from the mountain of genomes generated by sequencing [180]. Annotation-scoring schemes for automatic annotation methods are needed to allow the reduction of the cost of genome annotation. One proposed method that uses a genome comparison approach is the annotation confidence score (ACS) [181]. ACS attempts to combine sequence and textual similarity to denote the quality of annotation. The quality score is derived from a summary of sequence homology, taxonomic distance, and textual similarity analysis. Another example is a semi-automated genome annotation comparison and integration scheme [182]. This scheme compares annotations and arrives at a consensus annotation based on the comparison outcome. An automated tool for Bacterial Genome Annotation Comparison (BEACON) [183] similarly enables a fully automated, simple, and quick comparison of genome annotations generated by multiple annotation methods. It yields analytical results that are comprehensive and informative. Functional annotation of prokaryotic genomes obtained by different annotation methods can be compared in BEACON, and it can be used to combine and extend annotations from different annotation methods.

Annotation edit distance (AED) is a different measure for annotation comparisons, which aims to evaluate changes across annotation releases [184]. It was introduced in 2009. It complements a similar measure, called Annotation Turnover, which tracks the addition and deletion of gene annotations between releases. AED determines structural changes to an annotation, such as alternative splicing, which cannot be reported by using conventional measures, such as sensitivity and specificity. AED is used as a quality-control measure in MAKER2, with some adaptation. MAKER2 uses AED to show alignment between a gene and the supporting evidence used. An AED of 0 in MAKER2 indicates a perfect match between the intron–exon coordinates of annotation and the used evidence, such as EST, protein, and mRNA-seq data. On the other hand, an AED of 1 indicates no evidence-based support. As implemented in MAKER2, AED can be used as a quality-measure tool. This was confirmed by investigations of the annotations of human and mouse genomes from RefSeq, which revealed an agreement between AED scores and domain contents in Pfam. In addition, the International Nucleotide Sequence Database Collaboration (INSDC) [185] has designed quality control procedures to be used in annotation pipelines, such as NCBI’s PGAP. The quality control matrices within PGAP are generated automatically, facilitating annotation submission to GenBank.

RefSeq is a highly curated collection of annotated genomes and transcripts that is widely used as a reference for genome projects and different analysis tools and is considered to contain high-quality annotations [186]. It is a collection of comprehensive and non-redundant, explicitly linked genomes, transcripts, and protein sequence records, with publications, informative nomenclature, and standardized and expanded annotations available. Quality assurance checks for different data types are applied to all RefSeq data. This renders the RefSeq data consistent and allows it to serve as a baseline for multiple gene-specific reporting and cross-species comparisons. For annotation, the RefSeq dataset uses both computational methodology and manual curation by NCBI scientific staff. The RefSeq dataset is freely accessible. Its 201st release contains data on more than 103,000 organisms and can be accessed using NCBI’s nucleotide and protein databases, BLAST databases, and through FTP.

## 7. Re-Annotation and Future of Annotation

### 7.1. Re-Annotation

We have seen that as a result of the increasing volume of data from genome sequencing projects, computational analysis methods have become a considerable element of genome annotations. However, this has led to high levels of misannotation in public databases [187,188]. Since annotations are used as a resource in other annotation projects, researchers have to be presented with high-quality data. To ensure such high-quality data, NCBI and other sequencing centers have developed international annotation standards [189]. While re-annotation is crucial for correcting some missannotations [190], other main motivations for re-annotation are the discovery of new genes or protein functions, comparison of new and existing annotation methods, and assessment of annotation reproducibility [191]. Re-annotation benefits the end-user by providing the latest resources. Updating and re-annotating genome annotations is necessary for the provision of accurate and relevant information, because the knowledge of gene products is expanded each day by downstream research, such as comparative genomics, transcriptomics, proteomics, and metabolomics. Updating a previously annotated genome can be seen as re-annotation [192]. Automated annotations save time and resources, but manual annotations, although time-consuming, are better than automated annotations. Hence, aggregating and comparing multiple automated annotations side-by-side, followed by manual curation, will greatly reduce subsequent error propagation. Annotations of many of the first-generation genomes published were limited because of limited information and small numbers of references. Currently, because of the falling cost of sequencing, new assemblies, other evidence (such as RNA sequencing), and other genome technologies, these genomes are being re-annotated and updated [193,194,195].

In some ways, comparative studies are becoming more difficult because of the diverse annotation strategies and updates. Re-annotation can be used to create large complete genomes, and indeed, there are tools that can be used for this purpose. Restauro-G [196] is rapid bacterial genome re-annotation software that utilizes a BLAST-like alignment tool for re-annotation. MAKER2 incorporates an external annotation pass-through mechanism that accepts pre-existing genome annotations and aligned experimental evidence in GFF3 format as an input. This mechanism allows annotations from reference genomes to be done over the legacy annotation and creates a non-redundant consensus dataset after merging. Although annotations must be recomputed using the latest software and databases, there are no standard means to do this.

Yet another proposed approach is the Wiki solution, which is an open-editing framework for websites and data, where anyone can edit a shared resource [197]. Wiki-based sites have been proven successful in providing accurate, useful, and updated information, despite the fear of being filled with unreliable and inaccurate data. Currently, new information emerges from different corners of bioinformatic fields, which impacts gene annotation, rendering re-annotation a never-ending process, to some degree.

### 7.2. The Future of Annotation

The scope of genome annotation has expanded since the first complete annotation of the *Haemophilus influenzae* genome in 1995 [198]. The scope has widened to include information about noncoding RNAs [199], promoters and enhancers [200], pseudogenes [201], and many other features. Annotation will keep expanding, as the sequencing technology and knowledge related to genomics continues to evolve. Direct sequencing of RNA using Oxford Nanopore technology has been recently introduced [202]. Indeed, long-read RNA sequences improved human poly(A) RNA isoform characterization and allele specificity analysis. The method addresses the loss of information in high-throughput complementary DNA sequencing, which frequently copies biological RNA as short reads. Although the nanopore RNA sequencing technology is in its infant stage, it may soon allow low-cost sequencing of full transcripts. It could thus play a major role in future annotations [203]. As another suggestion for the future of annotation [204], the standard automated annotation practice relies on the majority rule that follows "the sequence tells the structure tells the function" stance, which hinders progress, because it generates and propagates errors. This inductive process discourages the discovery of novelty. It therefore argues for annotation systems that support multiple models of inference, such as deductive and abductive (trial and error method) inferences, in addition to the inductive processes used.

A different perspective on the future of annotation is the anticipation of multiple dimensions in characterizing genome-scale function [205]. From this perspective, identification of genes and assigning their functionality is considered to be a one-dimensional genome annotation, while specifying the cellular component and their interactions is a two-dimensional annotation. Three-dimensional annotation considers the effects of cellular packing and localization, i.e., the intracellular arrangement of chromosomes and other cell components. A fourth dimension would be the investigation of changes driven by adaptive evolution. While only one- and two-dimensional annotations are currently feasible, the higher-dimension annotations listed above and beyond should be possible in the future.

A popular computational approach that can be applied in annotation is machine learning. Machine learning constructs a mathematical model for a specific concept and identifies data patterns. This property is useful for genome annotation. Machine learning has already been implemented in finding functional elements in the human genome via unsupervised learning [206]. Machine-learning methods can be used to integrate multiple and heterogeneous datasets by applying complex functions, but the lack of training examples and the context specificity of models poses some challenges [207]. Although annotation tasks, such as protein function predictions [208] are challenging for machine-learning models, these models are likely to play a big role in future annotations considering the constant increase in the available data.

## 8. Conclusions

Understanding the structure of a gene is a crucial step in comprehending its function and the significance of variations. Computational annotation approaches, such as ab initio and homology-based annotations, enable such endeavors to be carried out automatically. Using automatic annotation systems and pipelines is imperative, considering the large amounts of sequence data generated by NGS. The importance of genome annotation ranges from answering in-depth questions about evolution to current applications, such as diagnosis of genetic disorders and drug design. This necessitates annotation quality-control, as errors can be easily propagated downstream. Quality-control methods and community annotations will help in avoiding such errors. Further, re-annotation is needed to correct faulty annotations or to update older annotations and even in some cases of well-studied genes, to identify novel features that were missed by preceding technologies. These factors frame annotation as an incessant journey, as new perspectives and technologies emerge every day.

## Figures and Tables

**Table 1:** Commonly used gene prediction programs.

Method	Program	Description	URL	Ref
Ab initio	EasyGene	HMM-based automatic gene predictor for prokaryotes that ranks open reading frames (ORFs) by statistical significance	https://services.healthtech.dtu.dk/service.php?EasyGene-1.2	[25]
	FGENESH	HMM-based gene structure prediction	http://www.softberry.com/berry.phtml?topic=fgenesh&group=programs&subgroup=gfind	[26]
	GeneMark	A family of self-training gene prediction programs for bacteria, archaea, metagenomes, metatranscriptomes and eukaryotes	http://opal.biology.gatech.edu/GeneMark/	[27]
	GeneZilla	Generalized hidden Markov model (GHMM) eukaryotic gene finder (formerly known as TIGRscan)	http://www.genezilla.org/	[28]
	GenScan	Algorithm for ab initio prediction of complete gene structures in vertebrate, *Drosophila*, and plant genomic sequences	http://hollywood.mit.edu/GENSCAN.html	[29]
	GlimmerHMM	GHMM-based eukaryotic gene finder that incorporates splice sites from GeneSplicer and decision tree from GlimmerM in Unix environment	http://ccb.jhu.edu/software/glimmerhmm/	[30]
	HMMgene	HMM-based gene predictor for vertebrates and *C. elegans*, full as well as partial genes	https://services.healthtech.dtu.dk/service.php?HMMgene-1.1	[31]
	mGene	Web service for predicting eukaryotic gene structures, including protein-coding genes and untranslated region (UTR) with pre-trained models	https://galaxy.inf.ethz.ch/tool_runner?tool_id=mgenepredict	[32]
	NetGene	Predicts splice sites in human, *C. elegans* and *A. thaliana* DNA	https://services.healthtech.dtu.dk/service.php?NetGene2-2.42	[33]
	RNAmmer	A two level HMM-based predictor of rRNA genes in full genome sequences	http://www.cbs.dtu.dk/services/RNAmmer/	[34]
	SNAP	Semi-HMM general-purpose gene finding program suitable for both eukaryotic and prokaryotic genomes	https://github.com/KorfLab/SNAP	[35]
	tRNAscan-SE	A covariance model-based program that provides genomic coordinates, predicted function, and secondary structure of tRNA genes	http://lowelab.ucsc.edu/tRNAscan-SE/	[36]
Homology	GeMoMa	A program that uses annotated genes to infer protein-coding genes in a target genome	http://galaxy.informatik.uni-halle.de/	[37]
	GenomeThreader	Uses cDNA, EST and protein sequences to predict gene structures via spliced alignments	http://genomethreader.org/	[38]
	PPFINDER	Identifier of processed pseudogenes incorporated in mammalian genome annotation	https://mblab.wustl.edu/software.html	[39]
	PseudoPipe	A computational pipeline that searches a mammalian genome and identifies pseudogene sequences	http://www.pseudogene.org/pseudopipe/	[40]
	TWINSCAN	GenScan extension, gene structure prediction system that exploits homology of related genomes	https://mblab.wustl.edu/software.html	[41]
Combined	AUGUSTUS	An ab initio gene prediction program that can also incorporate extrinsic sources, e.g., EST alignment, protein alignments and syntetic genome alignments	http://bioinf.uni-greifswald.de/augustus/	[42]
	JIGSAW	Gene model predictor that combines outputs from other gene finders, splice site predictors, and sequence alignments	http://www.cbcb.umd.edu/software/jigsaw/	[43]

**Table 2:** Ontology based annotation tools.

Program	Description	URL	Ref
BLAST2GO	A comprehensive bioinformatics tool for functional annotation of sequences and data mining on annotation results	https://www.blast2go.com/	[70]
FastAnnotator	An integration of well-established annotation tools for annotation of transcripts, which assigns GO terms, enzyme commission numbers, and functional domains	-	[71]
GO FEAT	Homology-based functional annotation tool for genomic and transcriptomic data	http://computationalbiology.ufpa.br/gofeat/	[72]
GOtcha	A method that predicts gene product function by annotation with GO terms	http://www.compbio.dundee.ac.uk/gotcha/gotcha.php	[73]
PANNZER2	A fully automated service for functional annotation of prokaryotic and eukaryotic proteins of unknown function that provides both GO annotations and free text description predictions	http://ekhidna2.biocenter.helsinki.fi/sanspanz/	[74]
PoGO	A statistical pattern recognition method that assigns GO terms for fungal proteins	-	[75]

**Table 3:** Ab initio and Homology-based annotation tools summary.

	Gene Prediction	Source of Data	Evolutionary Distance Effect	Strength
Ab initio	Rely on statistical model and gene signal	Models (HMM, GHMM, WAM) that can be trained supervised or unsupervised	Medium	Fast and easy means to identify and novel genes
Homology	Rely on sequence alignment	Proteins, EST, cDNA	High	Better accuracy, suitable for functional annotations
